# Phase II Study of Hypofractionated Proton Beam Therapy for Hepatocellular Carcinoma

**DOI:** 10.3389/fonc.2020.00542

**Published:** 2020-04-28

**Authors:** Tae Hyun Kim, Joong-Won Park, Bo Hyun Kim, Eun Sang Oh, Sang Hee Youn, Sung Ho Moon, Sang Soo Kim, Sang Myung Woo, Young-Hwan Koh, Woo Jin Lee, Dae Yong Kim

**Affiliations:** ^1^Center for Liver Cancer, Research Institute and Hospital, National Cancer Center, Goyang, South Korea; ^2^Center for Proton Therapy, Research Institute and Hospital, National Cancer Center, Goyang, South Korea

**Keywords:** hepatocellular carcinoma, overall survival, local progression-free survival, proton beam therapy, radiotherapy

## Abstract

**Background:** Proton beam has an excellent depth dose distribution due to its unique physical properties, and thus proton beam therapy (PBT) has been tried and showed promising outcomes in hepatocellular carcinoma (HCC). The purpose of this phase II study is to evaluate the efficacy of hypofractionated PBT in HCC.

**Methods:** The eligibility criteria for this study were as follows: patients with HCC lesion(s) who were failed after, were difficult to treat with, or refused to other local treatments; tumor size and number of ≤7 and ≤2 cm, respectively, and HCC lesion(s) of ≥2 cm from gastrointestinal organs; Child–Pugh score of ≤7; Eastern Cooperative Oncology Group performance status ≤1; and age ≥18 years. The prescribed dose of PBT was 70 Gy equivalent in 10 fractions. The primary endpoint was 3-year local progression-free survival (LPFS) rate.

**Results:** Forty-five patients were prospectively enrolled, and there were 35 men and 10 women with a median age of 63 years (range, 46–78 years). Thirty-seven patients had recurrent and/or residual disease, and eight patients had treatment-naive disease. All patients received the planned treatments without treatment interruption, and grade ≥3 acute toxicity did not occur. The median follow-up duration was 35.1 months (range, 11.2–56.3 months) and local progression occurred in two patients (8.7%). The 3-year rates of LPFS and overall survival (OS) were 95.2% (95% confidence interval [CI], 89.1%−100%) and 86.4% (95% CI, 72.9–99.9%), respectively.

**Conclusion:** Hypofractionated PBT showed promising LPFS and OS, and further studies are warranted to compare PBT with other local modalities.

## Introduction

Hepatocellular carcinoma (HCC) patients mostly have an underlying chronic liver disease resulting from hepatitis B (HBV) and C virus infection, alcoholic liver disease, nonalcoholic fatty liver disease, and so on. Intrahepatic disease progression is the main cause of death in nonmetastatic HCC patients (1). Thus, effective local treatments in these patients are crucial. Various local treatment options for HCC patients, such as surgical resection, liver transplantation, local ablative treatments including thermal ablation and percutaneous ethanol injection, transarterial chemoembolization (TACE), radioembolization, and so on, have been available (2–5), but many factors including tumor burdens, tumor characteristics, underlying liver function, and patient comorbidities limit the treatment options.

With recent technological advances in radiotherapy (RT) and biologic understanding of liver tolerance to RT, modern sophisticated RT techniques, such as three-dimensional conformal RT, intensity-modulated RT, and stereotactic body RT (SBRT), have made it possible to deliver high doses of radiation to tumor(s) and reduce radiation doses to surrounding noncancerous tissues including the remaining normal liver and gastrointestinal (GI) organs, and these have shown promising outcomes in HCC patients with/without tumor vascular thrombosis (TVT) (6–9). Compared with RT with X-ray, proton beam therapy (PBT), due to the inherent physical properties of the proton beam (called Bragg peaks), has an excellent depth dose distribution, which can increase the dose to the tumor while maintaining the radiation dose in the noncancerous portion of the liver (10–12). Recently, PBT with various fractionations (i.e., 4–34 fractions) has been attempted and showed encouraging outcomes (13–24). Theoretically, hypofractionated RT can potentially improve the therapeutic ratio compared with conventional fractionated RT by reducing the cancer cell proliferation within the tolerances of surrounding noncancerous tissues and shortening the overall treatment time. Based on this rationale, this single-institutional, single-arm, prospective study was conducted to evaluate the efficacy of hypofractionated PBT for HCC patients.

## Materials and Methods

### Patients

Patients were enrolled in a prospective clinical trial. The eligibility criteria for this study were as follows: (i) HCC was diagnosed with pathological confirmation or radiologic findings and serum α-fetoprotein concentrations of ≥200 ng/mL based on the guidelines of the Korean Liver Cancer Study Group and the National Cancer Center (NCC) (3); (ii) patients with primary or recurrent HCC lesions who were failed after, were difficult to treat with, or refused to other local treatments including surgical resection, radiofrequency ablation (RFA), TACE, and so on; (iii) the largest diameter and number of target lesion(s) were ≤7 and ≤2 cm, respectively, and targeted lesion(s) were ≥2 cm from GI organs; (iv) no history of prior RT to targeted lesion(s); (v) no evidence of extrahepatic metastasis; (vi) Child–Pugh score of ≤7 without uncontrolled ascites; (vii) Eastern Cooperative Oncology Group performance status of ≤1; (viii) age of ≥18 years; and (ix) adequate bone marrow (white blood cell count 1,500/mm^3^, platelet count 30,000/mm^3^, and hemoglobin 7.5 g/dL) and liver (total bilirubin ≤3.0 mg/dL, and aspartate aminotransferase and alanine aminotransferase <5.0 × upper limit of normal) functions. Clinical and tumor stage was classified by the Barcelona Clinic Liver Cancer (BCLC) (5) and the Modified Union for International Cancer Control (mUICC) (25) staging classification, respectively. This study was approved by the institutional review board of NCC (NCC20150042) and registered at www.clinicaltrials.gov (NCT02395523). Written informed consent was obtained for all patients before enrollment.

### Treatment

The simulation, plan, and treatment procedures of PBT have been previously reported (11, 13–15, 17, 18). Briefly, a four-dimensional computed tomography (CT) scan with contrast was performed in all patients under monitoring the respiration signals by a real-time position management (RPM) system (Varian Medical Systems, Palo Alto, CA, USA), and all obtained CT images were resorted into 10 equally spaced respiratory phases. The gross tumor volume (GTV) was delineated in the average intensity projection CT images, reconstructed with the CT images of gated (exhalation) phases (30% of total respiratory cycle). The internal target volume (ITV) was determined as the sum of the GTVs without margin from GTV for clinical target volume (11, 13–15, 17, 18, 21, 26), and the contours of the organs at risk (OARs) were delineated in each CT image during the gated phases. The planning target volumes (PTVs) included the ITV plus a margin of 5–7 mm in all directions. Typically, PBT plans (version 8.1; Varian Medical Systems) were performed with two to four (median of three) 230-MeV proton beams (Proteus 235; Ion Beam Applications, S.A., Louvain-la-Neuve, Belgium) using the double-scattering mode to design so that 100% of each prescribed dose would cover at least 95% of the PTV. The delivered irradiated doses of PBT to target and OARs were described in gray equivalents [GyE = physical dose of proton (in gray) × relative biologic effectiveness of proton (1.1)] and the equivalent dose in 2-Gy fractions [EQD2 (GyE_10_ or GyE_3_) = total dose × ({fraction dose+ α/β}/{2+α/β}), α/β of 3 and 10 for the late and acute effects, respectively] (27). The prescribed dose to PTV was 70 GyE (EQD2, 99.2 GyE_10_) in 10 fractions considering dose-fractionation regimens of our studies [50–72 GyE in 10–24 fractions (EQD2, 52.1–91.3 GyE_10_)] (13–18) and those of other studies [24–91 GyE in 6–30 fractions (EQD2, 28–103.5 GyE_10_)] (19–23). The dose volume constraints to the OARs were described in detail in our previous reports (11, 13–18, 28). The relative volumes of the remaining normal liver (total liver—GTV) and total liver receiving more than 27 GyE were limited to <50 and 60%, respectively, and the absolute volumes of the bowel and stomach receiving more than 35 and 37 GyE, respectively, were limited to <2 cm^3^. At each treatment, all patients were asked to fast for at least 4 h before treatment to diminish intrafractional and interfractional uncertainties. Each patient's position and isocenter were verified using digital orthogonal fluoroscopy, and radiation was delivered during the gated (exhalation) phases under monitoring the respiration signals by RPM system.

### Evaluation and Statistical Considerations

During PBT, patients were assessed weekly and, after completion of PBT, at the first month, every 3 months for the first 2 years, every 6 months up to 5 years, and yearly thereafter. Clinical, laboratory, and radiological examinations were performed at each follow-up. The tumor responses were assessed according to the modified Response Evaluation Criteria in Solid Tumors criteria (29) by comparing pre- and post-PBT CT/MRI scans, and the severity of adverse effects was graded using the Common Terminology Criteria for Adverse Events (version 4.0).

The primary endpoint of this study was local progression-free survival (LPFS), and we set the expected 3-year LPFS in patients treated with PBT at 80% or higher, considering results of our previous and other studies (17, 19, 22, 23, 30), with a threshold of 15%. With a power of 80%, a type I error level of 10% and a follow-up loss rate of 10%, 45 patients were required for enrollment. The definition of local, intrahepatic, and distant progression was a regrowth or new tumor within the PTV, within liver outside of the PTV, and extrahepatic sites, respectively. The times of LPFS, progression-free survival (PFS), and overall survival (OS) were determined from the commencement date of PBT to the date of local progression, disease progression or death, and death or last follow-up, respectively. Survival was estimated using the Kaplan–Meier method, and the difference in the survival curve was evaluated with the log-rank test in univariate analysis. Statistical significance was set to a *p* < 0.05, and all statistical tests were performed using STATA software (version 14.0; StataCorp, College Station, TX, USA).

## Results

**Figure 1:**
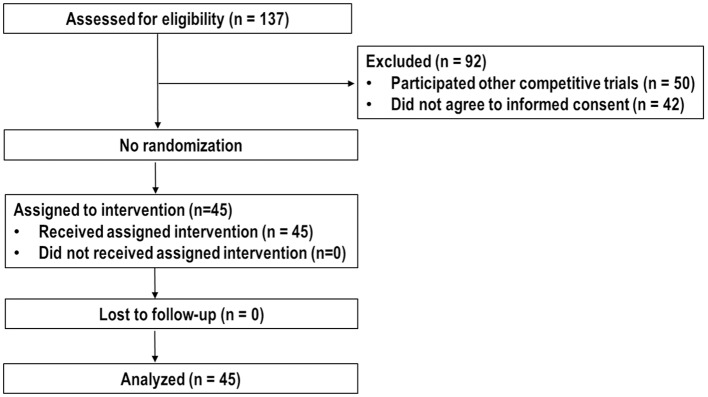
CONSORT diagram.

Between March 2015 and September 2018, 137 patients were assessed the eligibility for this trial. Of these, 50 patients participated in other competitive trials, 42 patients did not agree to inform consent, and the remaining 45 patients were enrolled and analyzed (Figure 1). Patient characteristics at the time of PBT are summarized in Table 1. Most (*n* = 37, 82.2%) patients, except eight patients (17.8%) who were treatment-naive due to difficult-to-treat lesions or refused to undergo other local treatments, had recurrent and/or residual tumor(s) in the PBT site, and the number of lesions treated with PBT was one and two in 42 and three patients, respectively. The median time of follow-up was 35.1 months (range, 11.2–56.3 months).

**Table 1:** Patient and treatment characteristics.

**Characteristics**		***n* (%)**
Gender	Male	35 (77.8)
	Female	10 (22.2)
Age, years	Median (range)	63 (46–78)
	<60	14 (31.1)
	60–69.9	23 (51.1)
	≥70	8 (17.8)
ECOG PS	0	45 (100)
Etiology of LC	HBV	38 (84.4)
	HCV	2 (5.7)
	Alcoholic	2 (5.7)
	Unknown	3 (6.7)
Child–Pugh classification	A	45 (100)
AFP, ng/mL	Median (range)	8.6 (0.6–5543.3)
	<10	24 (53.3)
	≥10	21 (46.7)
Tumor size, cm	Median (range)	1.6 (1.0–6.8)
	≤2	28 (62.2)
	>2	17 (37.8)
No. of treated lesions	1	42 (93.3)
	2	3 (6.7)
TVT	No	44 (97.8)
	Branch	1 (2.2)
mUICC stage	I	16 (35.6)
	II	24 (53.3)
	III	5 (11.1)
BCLC stage	A	34 (75.6)
	B	10 (22.2)
	C	1 (2.2)
Diagnosis at PBT	Primary	8 (17.8)
	Recurrence	37 (82.2)
Pre-Tx to PBT site	No	14 (31.1)
	Yes	31 (68.9)
	TACE	25 (80.7)
	RFA	5 (16.1)
	TACE + RFA	1 (3.2)
Pre-Tx to other sites	No	21 (46.7)
	Yes	24 (53.3)
	TACE	10 (41.7)
	SR	5 (20.8)
	SR + TACE and/or RFA	4 (16.7)
	TACE + RFA	3 (12.5)
	RFA	2 (8.3)
Planning target volume, cm^3^	Median (range)	17.9 (7.0–294.0)
Remaining normal liver (RNL) volume, cm^3^	Median (range)	1,175.8 (622.0–2,072.0)
_RNL_V_27GyE_, %	Median (range)	6.6 (3.6–17.6)
Total liver (TL) volume, mL	Median (range)	1,215.5 (646.0–2,121.0)
_TL_V_27GyE_, %	Median (range)	8.2 (4.3–34.6)
_Stomach_D_2cc_, GyE	Median (range)	0.0 (0.0–34.3)
_Esophagus_D_2cc_, GyE	Median (range)	0.0 (0.0–32.0)
_Duodenum_D_2cc_, GyE	Median (range)	0.0 (0.0–22.6)
_Bowel_D_2cc_, GyE	Median (range)	0.0 (0.0–17.5)
_Cord_D_2cc_, GyE	Median (range)	0.0 (0.0–31.3)

*LC, liver cirrhosis; HBV, hepatitis B virus; HCV, hepatitis C virus; AFP, α-fetoprotein; ECOG PS, Eastern Cooperative Oncology Group performance status; TVT, tumor vascular thrombosis; mUICC stage, modified International Union Against Cancer stage; BCLC stage, Barcelona Clinic Liver Cancer stage; Tx, treatment; PBT, proton beam therapy; TACE, transarterial chemoembolization; RFA, radiofrequency ablation; SR, surgical resection; _RNL_V_27GyE_, relative volume of the remaining normal liver receiving ≥27 GyE; _TL_V_27GyE_, relative volume of the total liver receiving ≥27 GyE; D_2cc_, delivered radiation dose to the stomach, esophagus, duodenum, bowel, and spinal cord of 2 cc (cm^3^); and GyE, gray equivalent*.

**Figure 2:**
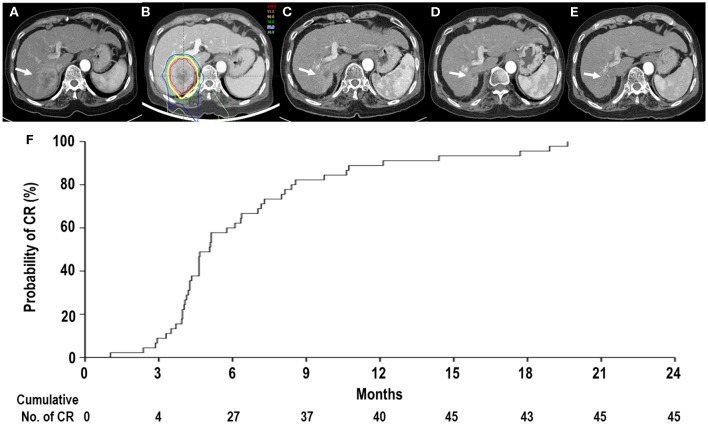
Tumor response after proton beam therapy (PBT). **(A)** Computed tomography scans prior to PBT showing the tumor (arrow). **(B)** The patient received PBT. **(C,D)** Computed tomography scans at 4 and 11 months, respectively, after PBT showing shrinkage of the tumor (arrow). **(E)** Computed tomography scans at 14 months after PBT showing complete response (CR) of the tumor (arrow). **(F)** The actuarial CR probability curves of tumors after PBT.

**Figure 3:**
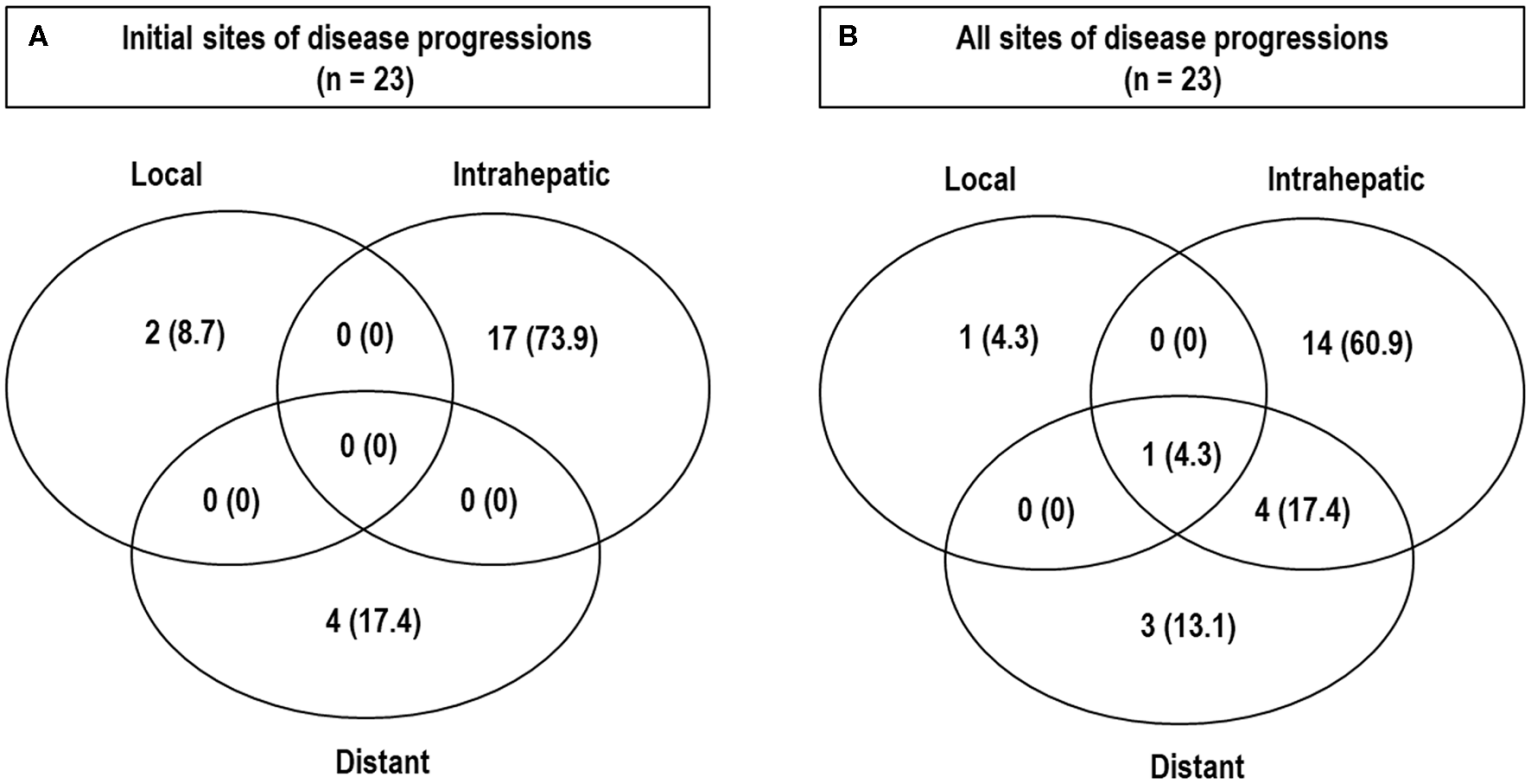
Patterns of disease progressions. Initial sites **(A)** and all sites **(B)** of disease progression at the time of analysis.

Of 45 patients, all treated lesions eventually reached complete response (CR), and the median time to CR was 5.1 months [95% confidence interval (CI), 4.3–6.3 months] (range, 1–19.6 months) (Figure 2). With increasing tumor size [2 cm (*n* = 28), 2.1–4 cm (*n* = 14), and >4 cm (*n* = 3)], the median times to CR increased [4.6 months (95% CI, 4.2–6.2 months), 6.4 months (95% CI, 4.0–9.7 months), and 5.1 months (95% CI, 5.1–10.6 months), respectively], but these differences were not significant (*p* = 0.675). At the time of analysis, 41 patients were alive, and four died of disease progression. Of 45 patients, disease progression occurred in 23 patients (51.1%) as follows: the initial sites of disease progression were local sites in two patients (8.7%), intrahepatic sites in 17 patients (73.9%), and distant sites in four patients (17.4%), and all of the sites of disease progression at the time of analysis were local sites in two patients (8.7%), intrahepatic sites in 19 patients (82.6%), and distant sites in eight patients (34.8%) (Figure 3). The median times to local, intrahepatic, and distant disease progression were 12.5 months (range, 10.5–14.4 months), 15.4 months (range, 2–30.1 months), and 20.6 months (range, 1.5–22.5 months), respectively. After disease progression was confirmed, 22 of 23 patients (95.7%), except for one patient due to poor performance status, were treated with salvage treatments, such as one or combinations of local and/or systemic treatments (i.e., surgical resection, RFA, TACE, PBT, RT, sorafenib, etc.).

**Figure 4:**
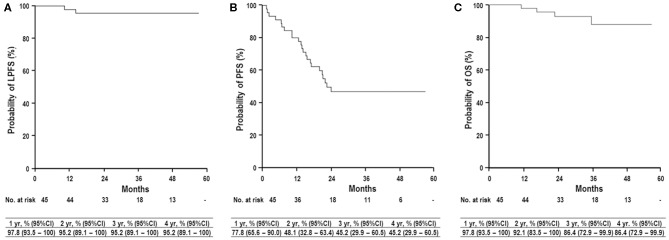
Local progression-free survival (LPFS) **(A)**, progression-free survival (PFS) **(B)**, and overall survival (OS) **(C)** curves in all patients. yr, year; CI, confidence interval.

The 3-year rates of LPFS, PFS, and OS were 95.2% (95% CI, 89.1%−100%), 45.2% (95% CI, 29.9%−60.5%), and 86.4% (95% CI, 72.9%−99.9%), respectively (Figure 4). All pretreatment characteristics were not significantly related to LPFS in the univariate analysis (Table 2). Patients with recurrent lesion(s), previous history of treatment to PBT site(s) or other site(s), and mUICC stage II/III had a trend toward lower PFS than those with primary lesion(s), no history of treatment to PBT site(s) or other site(s), and mUICC stage I, but these differences were not statistically significant in the univariate analysis because of the small number of study populations (*n* = 45) (*p* > 0.05 each) (Table 2). The patients with HBV had significantly higher PFS than the others in the univariate analysis (*p* < 0.05 each) (Table 2). All pretreatment characteristics were not significantly related to OS in the univariate analysis (*p* > 0.05 each).

**Table 2:** Univariate analysis of pretreatment characteristics for local progression-free survival (LPFS), progression-free survival (PFS), and overall survival (OS).

			**LPFS**		**PFS**		**OS**	
**Characteristics**		**No. of patients**, ***n***	**3 years (95% CI), %**	p^*^	**3 years (95% CI), %**	p^*^	**3 years (95% CI), %**	p^*^
Gender	Male	35	94.1 (86.3–100)	0.451	49.2 (32.0–66.4)	0.679	89.7 (78.5–100)	0.917
	Female	10	100 (100)		27.4 (0.0–59.2)		83.3 (53.5–100)	
Age, years	<60	14	92.9 (79.4–100)	0.600	50.0 (32.0–66.4)	0.590	91.7 (76.0–100)	0.663
	≥60	31	96.8 (90.5–100)		42.8 (23.7–76.3)		83.6 (64.4–100)	
Etiology of LC	HBV	38	94.5 (87.1–100)	0.532	50.9 (33.8–68.0)	<0.001	93.1 (83.9–100)	0.122
	Others	7	100 (100)		14.3 (0–40.2)		68.6 (32.1–100)	
AFP, ng/mL	<10	24	95.8 (87.8–100)	0.949	49.3 (27.7–70.9)	0.288	91.0 (79.0–100)	0.940
	≥10	21	95.0 (85.4–100)		40.6 (18.8–62.4)		81.7 (57.6–100)	
Tumor size, cm	<2	27	96.4 (89.5–100)	0.764	52.2 (32.0–72.4)	0.192	95.5 (86.9–100)	0.183
	≥2	17	94.1 (82.9–100)		35.3 (12.6–58.0)		74.9 (48.2–100)	
No. of treated lesions	1	42	95.1 (88.4–100)	0.698	43.4 (27.3–59.5)	0.566	85.5 (71.0–100)	0.588
	2	3	100 (100)		66.7 (13.4–100)		100 (100)	
TVT	No	44	95.3 (88.8–100)	0.827	43.8 (28.3–59.3)	0.369	86.2 (72.5–99.9)	0.773
	Branch	1	– (–) [100 (100)]^†^		– (–) [100 (100)]^†^		– (–) [100 (100)]^†^	
mUICC stage	I	16	93.8 (81.8–100)	0.534	56.3 (28.1–84.5)	0.272	100 (100)	0.171
	II/III	29	96.4 88.8–100)		39.9 (21.7–58.1)		80.6 (62.0–99.2)	
BCLC stage	A	34	93.9 (85.7–100)	0.422	43.7 (26.3–61.1)	0.885	85.9 (70.4–100)	0.835
	B/C	11	100 (100)		50.9 (19.0–82.8)		90.9 (73.8–100)	
Diagnosis at PBT	Primary	8	100 (100)	0.498	72.9 (40.6–100)	0.115	100 (100)	0.304
	Recurrence	37	94.3 (86.7–100)		38.8 (22.1–55.5)		82.8 (65.7–99.9)	
Pre-Tx to PBT site	No	14	100 (100)	0.325	63.5 (37.8–87.2)	0.126	100 (100)	0.140
	Yes	31	93.2 (84.0–100)		36.1 (17.7–54.5)		79.3 (59.3–99.3)	
Pre-Tx to other sites	No	21	95.2 (86.2–100)	0.921	59.6 (37.8–81.3)	0.061	88.9 (68.3–100)	0.307
	Yes	24	95.5 (86.9–100)		31.4 (10.8–52.0)		85.0 (68.9–100)	

*CI, confidence interval; CR, complete response; all others are the same as in Table 1*.

* *Log-rank test*.

† *Two years*.

All patients received the planned treatments without interruption of the treatment course. Within 3 months after PBT, of 45 patients, grades 1 and 2 elevated alanine aminotransferase without evidence of disease progression developed in 3 (6.7%) and 0 patients (0%), respectively, and the Child–Pugh score showed a 1-point decrease in two patients (4.4%) and no change in 43 patients (95.6%); 0 patients (0%) had a ≥1-point increase. Sixteen (35.6%) and one (2.2%) patients experienced grades 1 and 2 leukopenia, respectively, and 10 (22.2%) and 0 (%) experienced grades 1 and 2 thrombocytopenia, respectively. Thirteen patients (28.9%) experienced grade 1 dermatitis, and no patient experienced grade ≥2 or higher dermatitis. After 3 months from end date of PBT, late GI toxicity including ulcer and bleeding and late hepatic failure or death related to PBT had not occurred.

## Discussion

Curative treatments, such as surgical resection, liver transplantation, and local ablative treatments, including RFA, offering a 5-year OS rate of 50–70% (2–5, 31), have been applied for selected BCLC 0/A HCC patients. For BCLC B HCC patients who have inoperable large or multifocal tumors not suitable for curative treatments, TACE is recommended as a first treatment with an expected median survival time of >30 months and a 3-year OS rate of 40.4% (3–5, 31, 32). In our institutional cohort data (31), 3-year OS rates in mUICC I/II patients with Child–Pugh class A initially treated with surgical resection, liver transplantation, and RFA were 83, 94.5, and 100%, respectively, and 3-year OS rates in mUICC III patients with Child–Pugh class A treated with surgical resection, liver transplantation, and TACE were 56, 66.7, and 40.3%, respectively. In the present study, PBT was applied to HCC patients who were failed after, were difficult to treat with, or refused to other local treatments, such as surgical resection, RFA, and TACE, and resulted in 3-year OS rates of 86.4, 85.9, 100, 100, and 80.6% in all BCLC A, BCLC B, mUICC I, and mUICC II/III patients, respectively. Similarly, Fukuda et al. (20) reported that the 5-year OS rates in treatment-naive BCLC 0/A and B patients treated with PBT were 69 and 66%, respectively. Although direct comparisons among the studies are difficult because of different tumor burdens, patient characteristics, and selection bias, these results of PBT in BCLC A/B patients were comparable to those of surgical resection, RFA, and TACE. Although there is no randomized study of PBT comparing with other local treatments, these findings suggested that PBT could be considered as one of the treatment modalities for these patients.

Treatments for HCC depend on various factors including tumor factors (i.e., stage, location, size, number, and echogenicity of tumor), remaining liver function, availability of transplant donors, and patient comorbidities. Thus, as an alternative to conventional treatments including surgical resection, RFA, and TACE, RT including SBRT has been tried for HCC patients who have failed after, are difficult to treat with, or refuse to conventional treatment modalities (3, 6–8, 21, 33, 34). A recent pooled analysis of SBRT using 24–60 Gy in three to six fractions (EQD2, 48–114.8 Gy_10_; median, 83.3 Gy_10_) has shown a 3-year LPFS of 83.9% and a 3-year OS of 48.3% (7). Wahl et al. (8) analyzed patients receiving SBRT (*n* = 63) and RFA (*n* = 161) and reported that the 2-year LPFS and OS rates were not significantly different (83.8 vs. 80.2%, *p* > 0.05; and 46.3 vs. 52.9%, *p* > 0.05, respectively), but a recent analysis of the National Cancer Database data for treatment with SBRT (*n* = 296) and RFA (*n* = 3,684) showed that 5-year OS was superior in RFA compared with SBRT (29.5 vs. 19.3%, *p* < 0.01) (6). Proton beam therapy using 24–84 GyE in 4–34 fractions (EQD2, 28–103.5 GyE_10_) has also been tried for patients with inoperable or recurrent HCC and has shown promising 2- or 3-year LPFS rates of 75–96% and 2- or 3-year OS rates of 45.1–66% (13–24). Because results from a randomized study comparing PBT with SBRT are not available to date, whether PBT is truly equivalent or superior to SBRT or conventional treatment modalities for tumor control remains unanswered. However, our and others' dosimetric studies of PBT compared to RT with X-rays showed that PBT can reduce the irradiated liver volume at low to intermediate dose levels and subsequently may potentially decrease the risk of liver toxicity by allowing dose escalation for tumors (10–12). Meta-analysis showed a lower incidence of toxicity in PBT compared to RT with X-ray, with no significant difference in LPFS and OS (35), and Sanford et al. (36) showed PBT was superior to RT with X-ray for OS by less liver toxicity. A recent systematic quantitative review of 13 SBRT studies for the HCC patients by Ohri et al. (34) did not show improvement of local tumor control by dose escalation. In a recent analysis of the National Cancer Database for T1–2N0 HCC patients treated with PBT (*n* = 71) or SBRT (*n* = 918), PBT and EQD2 of >83 GyE_10_ improved survival compared with SBRT and EQD2 of <83 GyE_10_, respectively (33). Our phase I dose-escalation study (17) showed that at least 78 GyE_10_ would be needed for local tumor control, and our previous studies of HCC patients with TVT (*n* = 41) and with/without TVT (*n* = 243) receiving risk-adapted PBT with 50–66 GyE in 10 fractions (13, 15) consistently showed that LPFS was superior at EQD2 of ≥80 GyE_10_ compared with EQD2 of <80 GyE_10_. Although direct comparisons among the studies were difficult because of the heterogeneity of patient characteristics, different tumor loads, comorbidities, and selection bias, the EQD2 used in the present study (70 GyE in 10 fractions; EQD2, 99.2 GyE_10_) was at the high end of the range in the previous studies (EQD2, 28–103.5 GyE_10_) and yielded the high end of LPFS and OS without grade ≥3 toxicity (17, 19, 22, 23, 30). These findings implied that dose escalation with PBT may improve local tumor control and survival by minimizing the risk of toxicity.

The present study included patients who had a tumor size and number of ≤7 cm and ≤2, respectively, and/or 2 cm away from GI organs and Child–Pugh class A. Thus, further prospective and large-scale studies considering patients with large (>7 cm), multiple (≥3) tumors and/or close to GI organs and Child–Pugh class B/C are needed to define the role of PBT in HCC patients. Based on promising outcomes of PBT in present and previous studies (13–15), we conducted a prospective cohort study of HCC patients receiving PBT (NCC20180197) to comprehensively evaluate the role of PBT in HCC patients. Because of the lack of a randomized trial comparing PBT with current established treatments, surgical resection, RFA, TACE, and so on, it is still unclear to date whether PBT can truly achieve outcomes comparable to those treatments. However, Bush et al. (24), in preliminary results of a phase III trial comparing PBT with TACE, showed a trend toward superior local tumor control and lower toxicity for PBT compared with TACE. We conducted a phase III study comparing PBT with RFA for HCC patients with recurrent or residual disease (NCT01963429), and enrollment was completed and awaiting data maturation.

In conclusion, this study showed that PBT could achieve promising LPFS and OS similar to those of curative treatments in BCLC A/B HCC patients with Child–Pugh class A in our institutional cohort (31) and other studies (2–5, 31, 32), with minimal toxicity. Although further prospective large-scale studies of PBT for patients with unfavorable tumors (i.e., large and multiple tumors and close to GI organs) and poor liver function (i.e., Child–Pugh class B and C) are needed, our data suggest that PBT could be considered as one of the therapeutic options in HCC patients depending on the tumor burden and patient morbidities.

## Data Availability Statement

The datasets used and/or analyzed during the current study are available from the corresponding author upon reasonable request.

## Ethics Statement

The studies involving human participants were reviewed and approved by Ethical Committee of National Cancer Center (NCC) (NCC20150042). The patients/participants provided their written informed consent to participate in this study.

## Author Contributions

TK, J-WP, and BK: conceptualization. EO, SY, SM, Y-HK, and DK: data collection. TK and SK: formal analysis. TK, J-WP, BK, SW, and WL: investigation. TK: writing original draft. J-WP, BK, and DK: supervision. All authors: reviewing and approval the final version of the manuscript.

## Conflict of Interest

The authors declare that the research was conducted in the absence of any commercial or financial relationships that could be construed as a potential conflict of interest.
